#  Sequential oligodiacetylene formation for progressive luminescent color conversion *via* co-micellar strategy†
†Electronic supplementary information (ESI) available: Detailed experimental and calculation procedures and spectra. See DOI: 10.1039/c5sc04253d


**DOI:** 10.1039/c5sc04253d

**Published:** 2015-12-09

**Authors:** Liangliang Zhu, M. Tuan Trinh, Liyuan Yin, Zhiyun Zhang

**Affiliations:** a State Key Laboratory of Molecular Engineering of Polymers , Department of Macromolecular Science , Fudan University , Shanghai 200433 , China . Email: zhuliangliang@fudan.edu.cn; b Department of Chemistry , Columbia University , New York , New York 10027 , USA; c Department of Chemistry , National Taiwan University , Taipei 10617 , Taiwan

## Abstract

Design and construction of organic systems that can enhance internal quantum efficiency to fully make use of radiative decay during the excitation process is highly desirable for the development of new-generation emissive materials. In practice, to employ those materials with an effective tuning of triplet-state involved multicolor luminescence is quite a considerable issue. This work takes advantage of a diphenyl-diacetylene-based co-micellar nano-ensemble that can undergo a sequential photocrosslinking to form a corresponding trimeric oligodiacetylene and a dimeric oligodiacetylene. Their emissive bands individually cover the visible-light spectral region and such a controllable dual band characteristic can result in a progressive luminescent color conversion from light-green to white and finally to purplish blue. In addition, the oligodiacetylenes possess an ultrafast intersystem crossing characteristic with a small S_1_–T_1_ energy gap. The leading thermally activated delayed fluorescence and phosphorescence can be employed to achieve long-life broadband emissions without chromatic aberration in this single organic system, towards the creation of emitting materials with high quantum efficiency. The synthesis of the sub-components is simple and the co-micellar strategy is extremely straightforward. Such a material design and approach may find potential uses in the microfabrication of novel organic light-emitting diodes and sophisticated controllable optoelectronic devices.

## Abstract 2

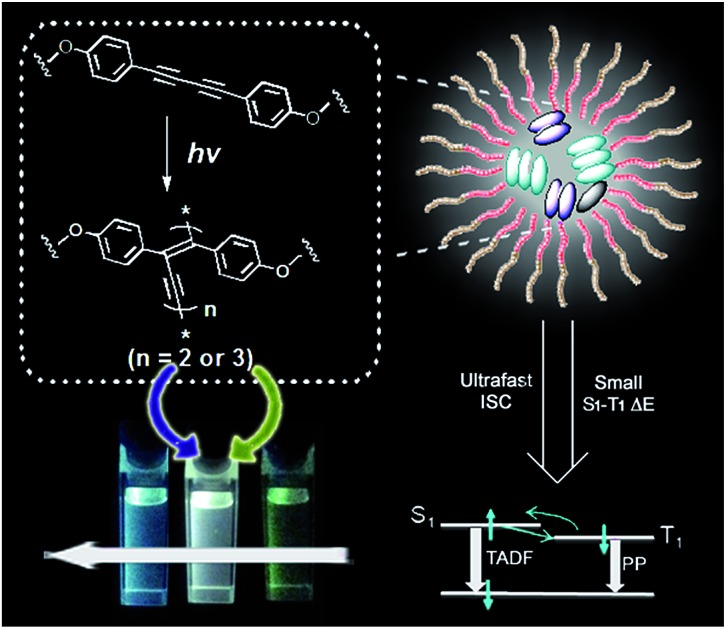
This work takes advantage of a diphenyl-diacetylene-based co-micellar nano-ensemble that can undergo a sequential photocrosslinking to form a corresponding trimeric oligodiacetylene and a dimeric oligodiacetylene.

## Introduction

Organic luminophores exhibiting strong visible-light emission are highly desirable for potential usage in numerous areas such as organic light-emitting diodes (OLEDs), biological imaging and chemosensing.^1^ In particular, rational designs displaying luminescent color conversion characteristics through external control can exhibit promising potential for fabrication of intelligent materials with variable emissive properties.^2^ Thus far, achieving the maximum radiative decay in these emissive materials has become increasingly preferable for practical application. Since a fluorescent emitter with singlet exciton formation has the limitation that only 25% of injected carriers can form emissive singlet excitons, the internal quantum efficiency can be effectively improved ideally up to 100% when both singlet and triplet excitons are involved in emitting light by enhancing intersystem crossing (ISC).^3^ In this way, we focused on the design and development of an organic system with triplet-state involved emission and a smartly tuning fashion for multicolor luminescence, which still remains a great challenge.

Diphenyl-diacetylene (DPDA) is a smart luminogen whose photophysics can be modulated upon self-assembly and photocrosslinking.^4^ Moreover, this luminogen undergoes significant triplet formation, potentially enabling long lifetime emissive applications.^5^ DPDA can be converted to its corresponding polymer species through a high ordered preorganization or form the corresponding oligomer species *via* a moderate aggregation, followed by thermal or photochemical reaction.^6^ However, to selectively form different oligomer species to make use of their synergic photophysical properties is very difficult to achieve. Interestingly, amphiphilic block copolymers are capable of a variety of self-assembly fashions (*e.g.* spherical micelles, cylinders, nanotubes, bilayers and lamellar phase), so as to serve a rich choice to form advanced nanomaterials or to direct the preparation of new types of functional materials.^7^ Herein, we demonstrate an optimized co-micellar strategy that takes advantage of non-covalent interactions between the well-selected organic luminophore and block copolymer template for realization of such a hypothesis.^8^ We expect that a selective formation of different oligodiacetylene species would emit triplet-state involved luminescence with different wavelength coverage to realize such an advanced intelligent material.

**Fig. 1:**
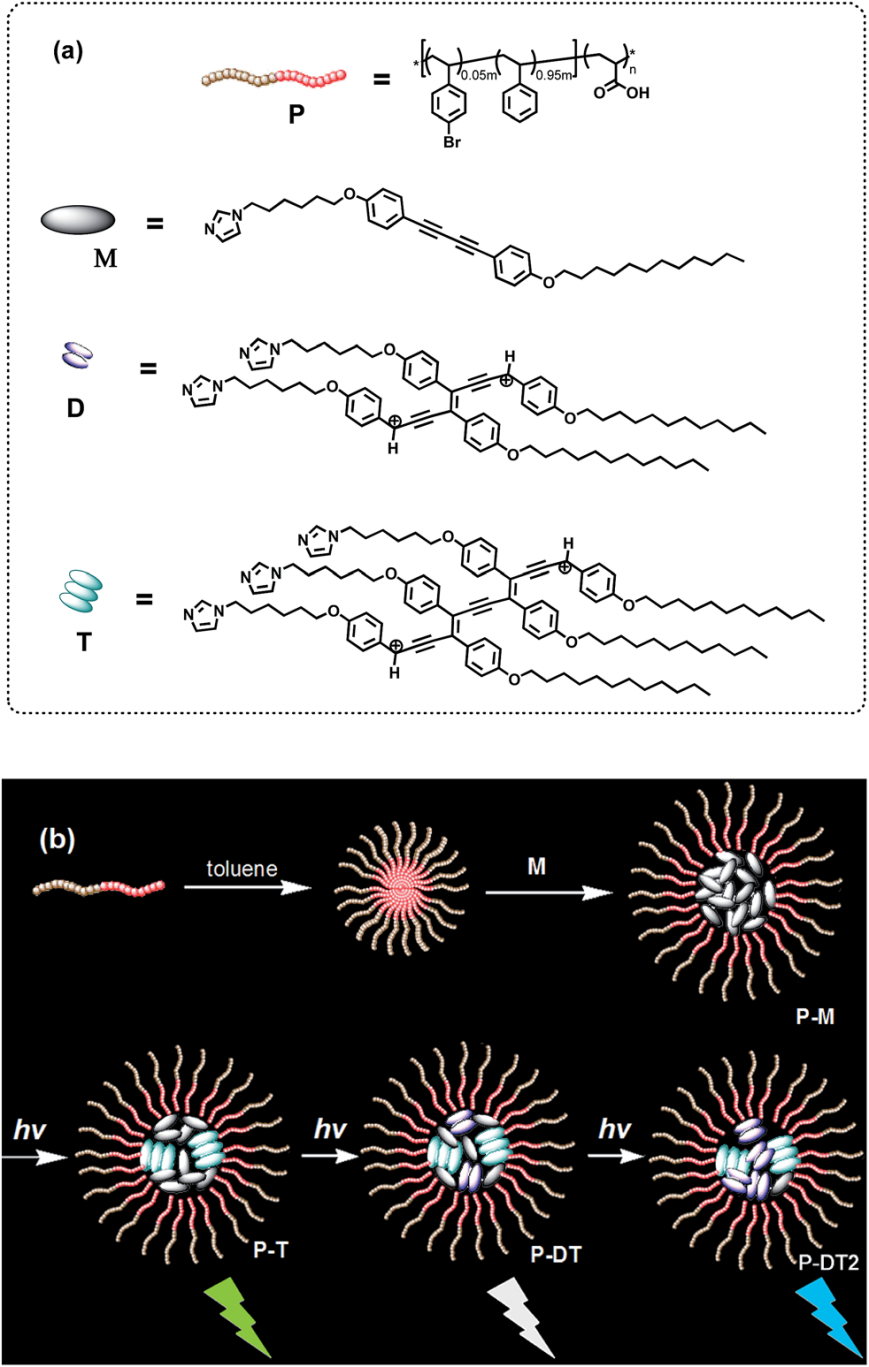
(a) Chemical structures of the block copolymer **P**, the diphenyl-diacetylene monomer **M** and the photoconverted oligo (diphenyl-diacetylene) of dimer D and trimer T. (b) A proposed process for the preparation route for the micellar ensemble **P-M** as well as the photoinduced luminescent color conversions of the co-micellar system: with an optimized encapsulation of the block copolymer, the trimeric oligodiacetylene will be preferentially formed in the ensemble first (P-T); continuous irradiation of the system further increases the content of the dimeric oligodiacetylene to generate P-DT and P-DT2. The luminescent color conversion originates from the change in the emissive proportion of the trimer and the dimer.

Thus, an amphiphilic block copolymer was made to serve as a micelle template in a certain solvent environment (compound **P**, see Fig. 1).^8^,^9^ Additionally, an imidazolyl modified diphenyl-diacetylene (DPDA) monomer with long alkyl chains was also designed and synthesized (compound **M**, see Fig. 1). Hydrogen-bonding interactions between the imidazolyl unit in **M** and the acrylic acid group in **P** were anticipated to construct the co-micelle nanostructures,^10^ while the introduction of long alkyl chains in **M** will also be favorable for the aggregate interactions inside the micellar core. Overall, we take advantage of such a supramolecular design to control the photocrosslinking degrees of DPDA as well as the emissive behaviors to achieve photoconversion of multicolor emission in a single co-micelle ensemble. The route for preparation of the supramolecular structure as well as the photocrosslinking process is outlined in Fig. 1 (see Experimental section and ESI† for the synthesis and preparation details).

## Results and discussion

To probe the strength of the H-bonding interaction between **M** and **P**, we investigated the association with a variation of the loading ratio (*R*, ranging from 0.1 to 1.5) of **M** relative to the acrylic acid units along the backbone of **P** by ^1^H NMR (Fig. S2†). This experiment was conducted in THF-d_8_ since the molecular self-aggregation can be minimized in this solvent to allow for the unassociated states of **M** and **P** (*vide infra*). As expected, peak broadening of the imidazolyl proton signals is exhibited at all *R* values, evidence of the association of **M** to the polymer backbone through H-bonding. These shifts reach a maximal downfield shift at *R* = 0.2 and then shift upfield with further increasing *R*. We attribute these changes to weakened H-bonding interactions between **M** and **P**, which may be due to steric crowding at high loading.^10^ On the other hand, we found that the photocrosslinking effect to form oligodiacetylenes (see more detailed discussion below) can also be weakened at high loading by monitoring the emissive band shift before and after irradiation in the free solutions (Fig. S3†). Thus, we focused on the loading ratio of *R* = 0.2 for the next co-micelle preparations.

**Fig. 2:**
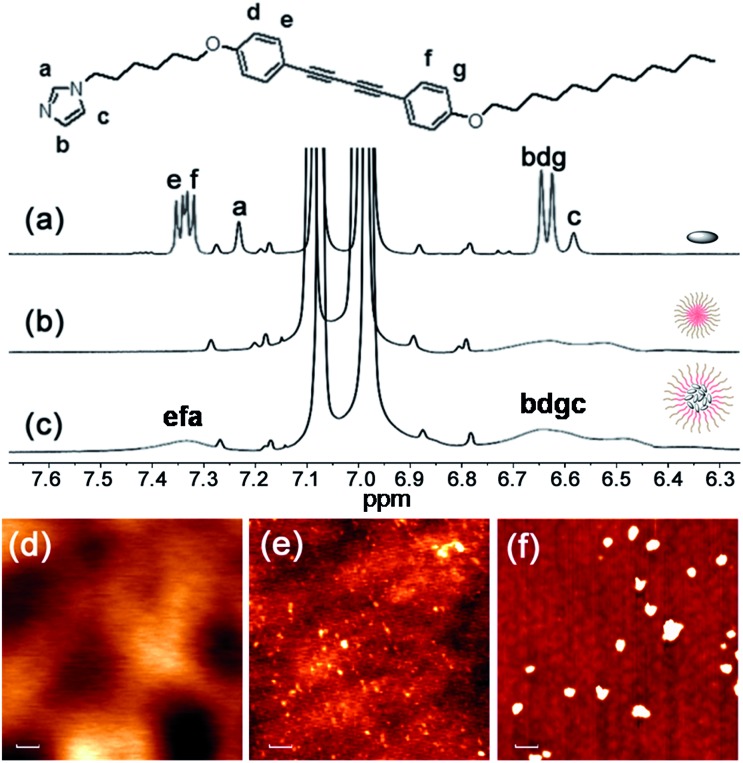
Characterizations of **P-M**: partial ^1^H NMR spectra (400 MHz, room temperature) of (a) **M**, (b) **P** and (c) **P-M** in toluene-d_8_. The tiny peaks without labels are assigned to satellite peaks from the solvent. AFM images of (d) **M**, (e) **P** and (f) **P-M** spun from toluene solution, respectively. Scale bar: 100 nm.

By optimization we found that toluene is the most favorable solvent for the co-micelle formation under our current experimental conditions. The block copolymer **P** can form a spherical micelle structure with a *Z*-diameter of over 20 nm in toluene (see the dynamic light scattering studies in Fig. S4†). As compared with Fig. 2a, all the aromatic protons of **M** turn quite broad in the presence of **P** (Fig. 2c), indicating the strong packing mode of **M** inside the micelle template. The formation of the co-micelle nanostructures can also be visualized by atomic force microscopy (AFM). As seen from Fig. 2d, it is difficult to distinguish the morphology of compound **M** since it shows liquid-crystal mesogenic properties in the solid surface.^11^ In contrast, the spherical micelle structure **P** and the co-micelle structure **P-M** can be verified by the observed dot-shape nanofeatures (Fig. 2e and f). We can even find that the average size of the co-micelle structures is bigger than the micelle **P** (see Fig. 2e and f and S4†). The increase of the size in the co-micelle structures, which can also be observed in a larger scale from scanning electron microscopy (SEM, Fig. S5†), further demonstrates the inclusion of **M** into the original micelle template. In total, these results suggest a co-micellar ensemble where the aggregated **M** was linked non-covalently with the inert poly(acrylic acid) group of **P**, leaving the polystyrene part of **P** stretched towards the toluene medium, as illustrated in Fig. 1.

**Fig. 3:**
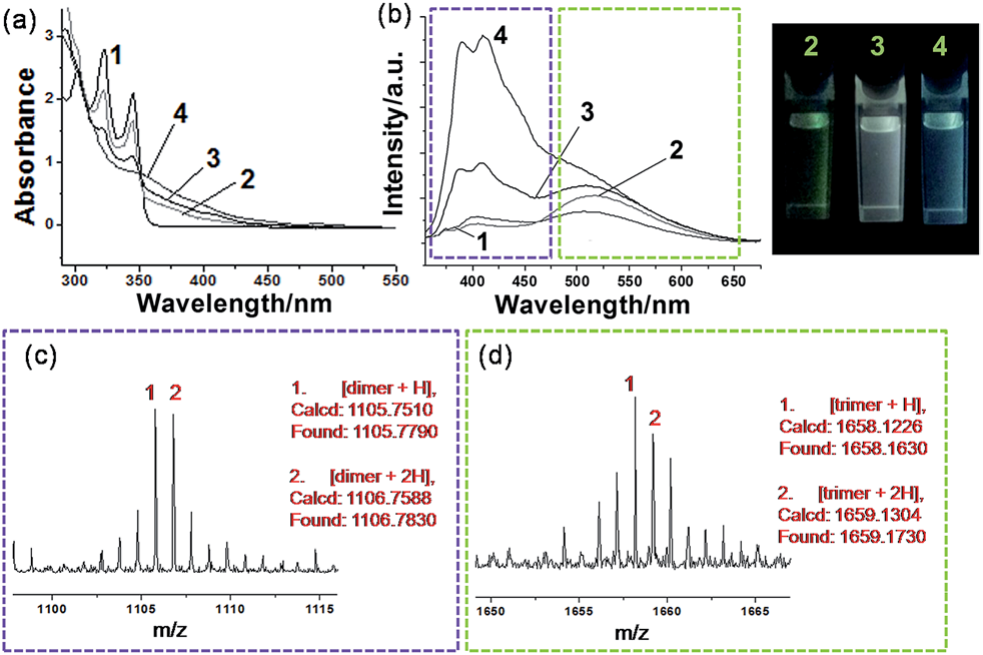
Sequential oligodiacetylene formation: (a) absorption spectra and (b) emission spectra upon 345 nm excitation of **P-M** in toluene at (1) initial state and after photoirradiation at 254 nm for (2) 5 min, (3) 15 min and (4) 30 min, and the photographs of the corresponding states 2, 3, and 4 under a UV light (*λ* = 365 nm). MALDI-TOF MS spectra of P-DT, prepared from **P-M** in toluene after photoirradiation at 254 nm for 15 min, showing (c) dimer signals and (d) trimer signals, respectively. All measurements were performed at room temperature.

Generally, photocrosslinking of diacetylenes cannot be conducted efficiently by a routine solution-based strategy. It has been widely accepted that molecular preorganization through template-grafting or controlled aggregation will promote this topochemical reaction.^12^ The photocrosslinking process (mainly a topochemical 1,4-addition reaction)^13^ here can be monitored by the absorption changes of the co-micelle system. Since there is no absorption band from the block copolymer **P** above 300 nm (see Fig. S6†), the absorption maxima at 302 nm, 321 nm and 345 nm originate from the alkynyl and aromatic chromophores of DPDA in **P-M** (see curve 1 of Fig. 3a). These absorption peaks were reduced upon UV irradiation, and the concomitant appearance of a continuous and broad absorption due to the oligodiacetylene conjugations was observed (curves 2, 3 and 4 of Fig. 3a).6a,c In the infrared spectrum of P-DT, the 2206 cm^–1^ vibration appeared due to the increased conjugation length along the triple bonds after photopolymerization (Fig. S7†), further demonstrating the formation of the enyne skeleton.6*b*

A more notable change upon irradiation was observed from the photoluminescence spectra. Herein, **P-M** reveals a relatively low emissive property in the initial state (curve 1 of Fig. 3b), probably due to the quenching effect of the long alkyl chain vibration and the self-aggregation of the chromophore. Upon irradiation at 254 nm, **P-M** can undergo a photocrosslinking process and presents strong broadband emissions (curves 2, 3 and 4 of Fig. 3b). The emission quantum yield for the photostationary state P-DT2 reaches ∼16.6% (relative to the standard Rhodamine B, *Φ* = 65%),^14^ as compared with ∼1.45% in **P-M**. The co-micelle size remains unchanged upon irradiation (see the DLS and AFM results in Fig. S8 and S9,† respectively), indicating that the photocrosslinking process of DPDA only takes place inside the micelle core. To our surprise, the emission change of the co-micelle exhibited a time-dependent dual-band behavior along with the photocrosslinking. An increase of the emission band at ∼520 nm (curve 2 of Fig. 3b), as well as a light-green luminescent solution, was first observed after the irradiation for 5 min. After 15 min of irradiation, the emission band around 400 nm was also enhanced (curve 3 of Fig. 3b) and the luminescent color of the solution gradually turned white. The continuous irradiation of the co-micelle solution led to further enhancement of the emission intensity at around 400 nm (curve 4 of Fig. 3b) and resulted in the solution luminescent color becoming purplish blue. With the encapsulation of the block copolymer, the molecular weight of oligodiacetylenes can be readily measured by matrix-assisted laser desorption/ionization (MALDI) time-of-flight (TOF) mass spectrometry. By monitoring the photostationary state of P-DT with MALDI-TOF, signals around *m*/*z* 1105 and 1658 were observed (Fig. 3c and d), which corresponded to the dimer and the trimer of the oligodiacetylenes, respectively. No signal from species of higher polymerization degree were observed (see the full spectrum in Fig. S10†). In this way, we here assumed that the dual luminescent bands in Fig. 3b originate from the emission of the dimer (∼400 nm) and the trimer (∼520 nm), respectively.

To validate this hypothesis, we performed a series of control experiments by employing compound **M** without the template **P**. When **M** was dissolved (or dispersed) in a variety of solvents, different photophysical behaviors can be observed upon irradiation. We can find similar emission enhancements at the wavelength of either 400 nm or 520 nm when **M** was placed in low or high polar solvents followed by irradiation (Fig. S11†).^15^ Specifically, the emission at 400 nm was significantly enhanced when **M** was placed in toluene for irradiation. The red shift of the monomer emission (relative to that in a moderately polar solvent) suggests that a self-aggregation of only **M** can also take place to some degree in toluene (Fig. S12†), allowing some degree of photocrosslinking. However, in this case, we only observe emission enhancement at 400 nm, not 520 nm. MALDI-TOF reveals that after irradiation in toluene, only the dimer signal (without the trimer signal) can be found in the spectrum of that photostationary state (Fig. S13†). This result and a comparison of the position and the shape of the emissions between Fig. S13† and 3b, allows us to assign the emission bands of ∼400 nm and ∼520 nm unambiguously to the dimer and the trimer, respectively, of the oligodiacetylenes in P-DT and P-DT2. For the co-micelle system, it would be reasonable that the trimer will be preferentially formed at the beginning to yield P-T since there are highly accumulated monomers in the core of **P-M**, followed by a further formation of dimer upon the dilution of the aggregated monomers inside the micelle to produce P-DT and P-DT2, as the illustrated process in Fig. 1 that is matched with the sequential dual-band characteristic in Fig. 3b. Interestingly, such a sequential oligodiacetylene formation process can also be visualized by MALDI-TOF MS signal change dynamics (Table S1 and Fig. S14†). We emphasize that this progressive photocrosslinking in this co-micellar structure can clearly yield the oligodiacetylene species with two terminal protons alongside the enyne skeleton (evidenced by [M + 2H]^+^ and [M + 2H]^2+^ signals in the MS spectra, see Fig. 3 and S15,† respectively), as shown in Fig. 1.

**Fig. 4:**
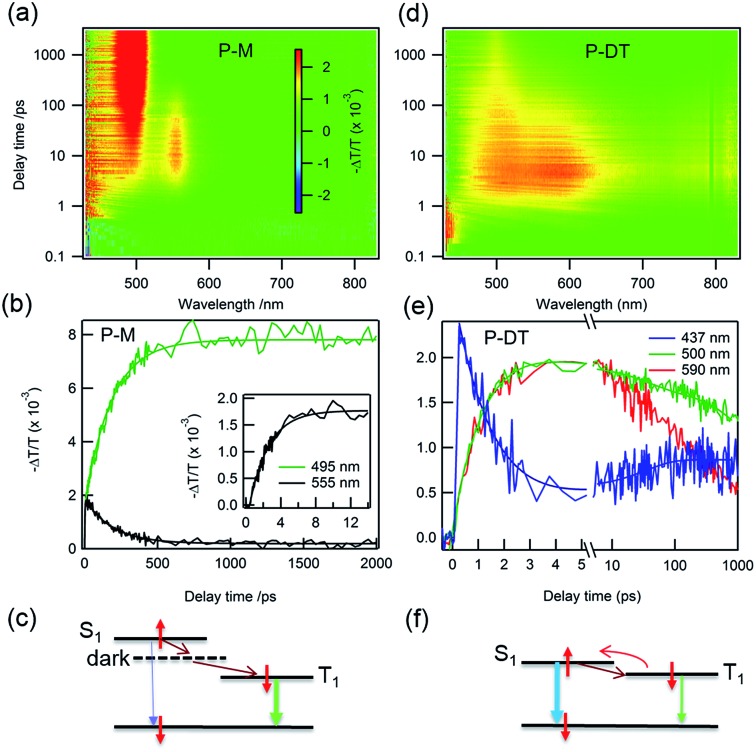
White-light supercontinuum fs-TA measurements. Differential transmission (–Δ*T*/*T*) as function of pump–probe delay time and probe wavelength for **P-M** pumped at 320 nm (a) and P-DT pumped at 375 nm (d). (b) Dynamics for **P-M** at 555 nm, dark state formation and decay, the inset is early buildup dynamics for the dark state, triplet formation at 495 nm (vertical cuts from figure a). (c) Schematic illustration of the processes in **P-M** after photo-excitation. (e) and (f): ultrafast intersystem crossing for P-DT. Dynamics for singlet (437 nm) and triplet (500 nm and 590 nm) excitons. All measurements were performed at room temperature.

Having characterized the co-micellar structure and the emission properties, we now turn to explore the triplet formation characteristics of these molecules using femtosecond transient absorption (fs-TA) spectroscopy. Fig. 4a shows the pseudocolor 2-D plot of differential transmission (–Δ*T*/*T*) as a function of pump–probe delay and probe wavelength for **P-M** upon photo-excitation at 320 nm. Since the linear absorption of **P-M** is in the UV range beyond the detection limit, we do not observe any bleaching from this molecule rather all observed signals are excited state absorption (ESA). There are two distinct ESA features centered at 495 and 555 nm. The ESA at 555 nm forms with a 2.2 ± 0.1 ps time constant (inset Fig. 4b) and decays with a time constant of 189 ± 5 ps. This decay coincides with the formation of the ESA signal at 495 nm (Fig. 4b). Since the ESA at 495 nm has a very long lifetime with no significant decay observed within the 2 ns time window, we assigned this ESA to triplet absorption from the lowest state (T_1_) to a higher lying triplet state (T_*n*_). The ESA at 555 nm cannot be optically excited and therefore is assigned as a dark state absorption. Fig. 4c summarizes the photo-physical processes in **P-M**. After photo-excitation singlet exciton S_1_ was created within the femtosecond time scale, S_1_ can decay back to S_0_ by emitting a photon or relaxing to a dark state in 2.2 ps. Excitons in the dark state then convert into triplet excitons with a 189 ps time constant. Triplet excitons will decay to the ground state *via* phosphorescence with a time scale of ms (see Fig. S16†). Note that the different ESA intensities for the triplet and the dark state excitons come from different absorption cross-sections. The singlet ESA is in the UV range and was not observed in this measurement.

**Fig. 5:**
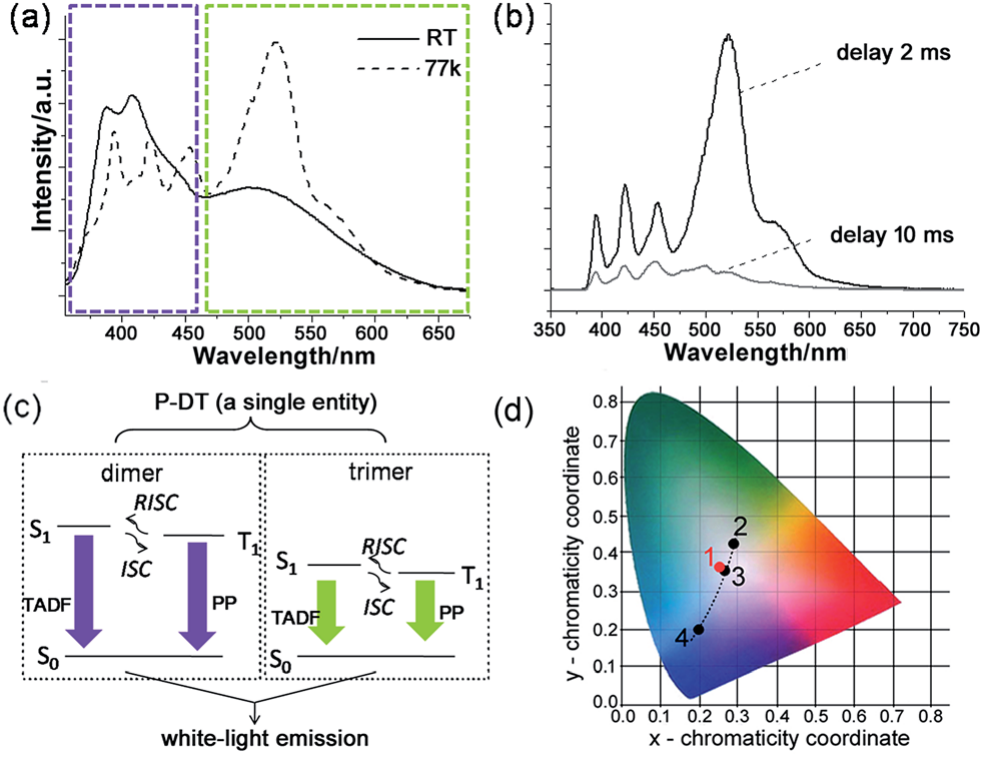
Broadband white-light emission of P-DT upon 345 nm excitation: (a) emission spectra of P-DT in toluene at room temperature (RT) and at 77 K. The purple and green-yellow dashed boxes here were marked to highlight the appearance of the bands at nearly the same wavelength. (b) Emission spectra of P-DT in toluene at 77 K with delay. (c) Proposed mechanism for the long-lifetime broadband emission of P-DT or P-DT2. (d) CIE 1931 chromaticity diagram. The red dot 1 signifies the luminescent color coordinate (0.25, 0.36) for the corresponding state in (a), while the black dots feature the luminescent color coordinates for the corresponding states in Fig. 3b, 2 (0.28, 0.42), 3 (0.26, 0.35) and 4 (0.19, 0.20).

There are dramatic differences in transient absorption spectra for P-DT. We observed three ESA features in the transient absorption spectra upon 375 nm excitation that are near the UV range (437 nm), at ∼500 nm, and at ∼590 nm (Fig. 4d). Note that there is no absorption for **P-M** at this excitation wavelength. A striking difference from the **P-M** molecule is that two ESA features in P-DT form at the same time with a much faster time constant of 1.4 ± 0.1 ps. The formation of two ESA features at 500 and 590 nm are at the same time constant with the decay of the signal at 437 nm (Fig. 4e). We assign the ESA at 437 nm to a singlet absorption which arises within the experimental resolution and the ESA at 500 nm to a triplet exciton in P-DT. The ESA at ∼590 nm might come from a dark state with an energy close to the triplet state of P-DT or a triplet state from a higher chain length oligomer. By pumping at different wavelengths (320 nm, 375 nm and 400 nm), we can easily figure out the triplet and singlet signal variations among the monomer, dimer and trimer (see the time independent TA spectra in Fig. S17†). Interestingly, the conversion from singlet to triplet excitons in P-DT is very fast. The singlet fission mechanism is energetically impossible here because the triplet state energy is too high,^16^ see Fig. 5 and ESI† for phosphorescence measurements. Therefore, the formation of the triplet here is only *via* intersystem crossing (ISC).

The time constant of 1.4 ps is remarkably fast for the ISC in organic molecules because of their weak spin–orbit coupling and a small singlet–triplet splitting. The ISC rate can be increased by introducing a heavy atom next to the delocalized π-system^17^ or reducing the singlet–triplet energy gap.^3^,^18^ Recently, Tilley*et al.* reported a rapid ISC for thionated perylene diimide (PDI) derivatives in which the rapid ISC was attributed to a very small singlet–triplet splitting.^19^ The rapid ISC in P-DT suggests a small singlet–triplet splitting and it also facilitates thermally activated delayed fluorescence, a reverse ISC process (Fig. 4f).^3^,^20^ Since the triplet state can be thermally activated back to the singlet state and it subsequently decays to the ground state *via* fluorescence, the triplet lifetime is therefore shortened. Fig. 4e shows the triplet ESA at 500 nm decays with two time constants of 30 ± 5 and 2400 ± 100 ps. The singlet ESA at 437 nm slightly increases with the time constant of 35 ± 10 ps, and decays with the same time constant as the triplet ESA after 100 ps (see Fig. S18†). The increase of the singlet ESA can be explained by the reverse ISC, which also suggests that more than one singlet state, *i.e.* from different oligomers, is involved in this process. By lowering the system temperature, we reduced the thermal activation energy and observed the coexistence of thermally activated delayed fluorescence (TADF) and phosphorescence (PP) (*vide infra*).

With understanding these natures, we turn to investigate the triplet-state involved emissions of P-DT. Although we have figured out the ultrafast ISC and reverse ISC mechanisms at room temperature, the long-lifetime emissions were still not easy to capture at room temperature due to various competitive kinetics. However, these factors can be significantly suppressed at low temperature to strengthen the long-lifetime emissions. As shown in Fig. 5a and b, the broadband emission behaved differently at 77 K with a different shape while compared with that at room temperature. The long lifetimes can also be observed by time-resolved emission measurements (see Fig. 5b and S19†). The excitation spectra from different emissions of P-DT reflect that the broadband emission at 77 K was still from a distinguished dimer/trimer emissive coverage (Fig. S20†). After a comparison of the broadband emission between at room temperature and at low temperature, we can find that there were no significant bathochromic-shifts for the emissions of both the dimer and the trimer, respectively (Fig. 5a). The appearance of the bands at nearly the same wavelength from 298 K to 77 K can further verify a small S_1_–T_1_ energy gap during the excitation of P-DT. To further analyze the broadband emission at low temperature, we investigated the luminescence spectra with different time delays. With a normalized approach to these spectra, it could be found that the emissive signals in the shorter wavelength region decayed faster than those in the longer wavelength region for both the dimer and the trimer bands (Fig. S21†). Since a TADF^3^ can be normally produced by reversed intersystem crossing from a small S_1_–T_1_ energy gap and appears hypsochromically with a shorter lifetime relative to the PP, the results here suggest the high energy set of peaks are from the dimer (TADF + PP) and the low energy set of peaks are from the trimer (TADF + PP) (see the proposed illustration in Fig. 5c). These two kinds of emissions simply have different lifetimes, resulting in some relative intensity change over time.

The luminescent color of the system upon the photocrosslinking can be accurately described through the Commission Internationale de l'Eclairage (CIE) diagram (see Fig. 5d). Since a white-light emission can be acquired by complementary-color or broadband emission,^21^ we can see the change in the emissive proportion of the trimer and the dimer would cause the systemic luminescent color to go from light-green to white and finally to purplish blue, along with the above-mentioned sequential oligodiacetylene formation process. Specifically, the CIE coordinate of the broadband fluorescence after 15 min irradiation was calculated to be (0.26, 0.35), which is quite close to the coordinate of the standard white-light illumination. Fine tuning for the warm and cold tints (green and purple parts) on it, respectively, can be commonly realized by prolonging the irradiation for different periods. Due to the small S_1_–T_1_ energy gap of the oligodiacetylenes, the wavelength of the broadband emission of P-DT did not change greatly at low temperature, allowing its luminescent color feature to remain close to the center of the white-light region (0.25, 0.36). In this way, white-light emissions at both room and low temperature can be achieved within this smartly designed co-micelle system. These white-light states can normally be maintained for hours to days even if the irradiation source is removed. The tunable luminescent color conversion and white light emission can also be observed in the **P-M** doped poly(methylmethacrylate) (PMMA) film (Fig. S22†), indicating such a co-micellar strategy controlled luminescence behavior can also work in the solid state.

## Conclusions

An oligodiacetylene-based co-micelle nano-ensemble was prepared by photo-induced crosslinking of the corresponding diphenyl-diacetylene monomer with encapsulation of a block copolymer template. The trimeric oligodiacetylene and the dimeric oligodiacetylene can be sequentially prepared through this co-micellar strategy. Their emissive bands individually cover the visible-light spectral region to form a tunable dual band emission in this single entity. A photoconversion of multicolor luminescence, including white-light generation, can be exclusively realized in this co-micelle system *via in situ* control.

Moreover, the oligodiacetylenes show an ultrafast ISC process and an extremely small S_1_–T_1_ energy splitting. Consequently, this material has triplet-state involved radiative decay (TADF and PP), allowing the long lifetime broadband white emission to be observed without chromatic aberration. As compared with the conventional blending techniques and heterogeneous approaches during the multicolor luminescence conversion process, the leading supramolecular system can serve as a novel single platform to provide such advanced optoelectronic properties. In addition, unlike the conventional diacetylenes with alkyl chains directly connected to the conjugated skeleton, the photophysical properties of the oligodiphenyl-diacetylenes are less sensitive to external interruptions. The current results could be valuable for the design of the next generation of OLEDs as well as advanced luminescent devices with smartly controllable manners.

## Experimental

### General

^1^H NMR and ^13^C NMR spectra were measured on a Bruker 400L spectrometer. The fast atom bombardment (FAB) mass spectra and high-resolution mass spectrometry (HR-MS) were recorded on a JMS-HX110 HF mass spectrometer (ionization mode: FAB+). Absorption spectra were recorded on a Shimadzu 1800 spectrophotometer, while the photoluminescent emission spectra were taken with a Jobin Yvon Fluorolog-3 spectrofluorometer (Model FL-TAU3). The photoirradiation was carried out using a PL Series compact UV lamp (4 W) with the irradiation wavelength of 254 nm. The distance between the lamp and the sample is kept within 3–5 cm. Time-resolved emission experiments were performed on an OB920 single-photo counting spectrometer. Transient absorption spectroscopy was conducted using a commercial Ti:Sapphire laser system (SpectraPhysics 800 nm, 100 fs, 3.5 mJ, 1 kHz). The atomic force microscopic (AFM) images were collected on a PSI XE100 Atomic Force Microscope (Park System). The scanning electron microscopic (SEM) images were obtained on a JSM 6340 scanning electron microscope (0.5–30 kV) equipped with a cold cathode field-emission gun (FEG) as the electron source. The hydrodynamic diameters of the micelles were measured by dynamic light scattering (DLS) using a Zetasizer Nano ZS system (Malvern). The photo images were photographed by a Nikon COOLPIX S8000 digital camera.

### Synthesis of compound **1**

The preparation for this compound was according to a similar procedure described in the literature.^22^

### Synthesis of compound **2**

A solution of dodecyl bromide (1.58 g, 6.36 mmol) in anhydrous acetone (5 mL) was added dropwise into a mixture of compound **1** (1.5 g, 6.41 mmol) and potassium carbonate (0.8 g, 5.8 mmol) in anhydrous acetone (15 mL) at 60 °C. The mixture was kept stirring for 12 h at that temperature under Ar protection. The solvent was removed *in vacuo*, and the residue was applied to silica gel chromatography (hexane/ethyl acetate = 7 : 1) to afford gray compound **2** (1.38 g, 53.5%). ^1^H NMR (400 MHz, CDCl_3_, 298 K): *δ* = 7.47 (d, *J* = 8.8 Hz, 2H), 7.44 (d, *J* = 8.8 Hz, 2H), 6.86 (d, *J* = 8.8 Hz, 2H), 6.81 (d, *J* = 8.4 Hz, 2H), 3.99 (t, *J* = 6.8 Hz, 2H), 1.81 (m, 2H), 1.48 (m, 2H), 1.30 (br, 16H), 0.92 (t, *J* = 6.4 Hz, 3H). ^13^C NMR (100 MHz, CDCl_3_, 298 K): *δ* = 159.90, 156.32, 134.28, 134.05, 115.65, 114.68, 114.35, 113.62, 81.41, 81.00, 73.01, 72.82, 68.18, 31.94, 30.28, 29.66, 29.65, 29.60, 29.58, 29.37, 29.16, 26.00, 22.71, 14.13. MS (FAB+): calcd for [M]^+^*m*/*z* = 402.3, found *m*/*z*: 402.3; HR-MS (FAB+): calcd for C_28_H_34_O_2_ [M]^+^*m*/*z* = 402.2559, found *m*/*z*: 402.2575.

### Synthesis of compound **3**

Compound **2** (764 mg, 1.9 mmol) was added with magnetic stirring to 1,6-dibromohexane (4.5 g, 18.4 mmol) acetone solution (5 mL). The mixture was then added to potassium carbonate (520 mg, 3.77 mmol). The mixture was stirred refluxing for 6 h under Ar protection and then was filtered. A great deal of solid was precipitated from the filtrate while it was added into a 4-fold volume of hexane. The solid was filtered, washed with petroleum (30 mL) and deionized water (20 mL), respectively, and dried under vacuum to obtain gray compound **3** (925 mg, 86.2%). ^1^H NMR (400 MHz, CDCl_3_, 298 K): *δ* = 7.47 (d, *J* = 8.8 Hz, 4H), 6.86 (d, *J* = 8.4 Hz, 4H), 4.00 (m, 4H), 3.46 (t, *J* = 6.8 Hz, 2H), 1.93 (m, 2H), 1.83 (m, 4H), 1.55 (m, 6H), 1.33 (br, 16H), 0.92 (t, *J* = 6.8 Hz, 3H). ^13^C NMR (100 MHz, CDCl_3_, 298 K): *δ* = 159.89, 159.75, 134.04, 134.04, 114.66, 114.64, 113.85, 113.67, 81.38, 81.26, 72.97, 72.88, 68.16, 67.85, 33.74, 32.66, 31.94, 29.67, 29.65, 29.60, 29.57, 29.37, 29.37, 29.15, 28.99, 27.91, 26.00, 25.27, 22.71, 14.13. MS (FAB+): calcd for [M]^+^*m*/*z* = 564.3 (^79^Br), found *m*/*z*: 564.3 (^79^Br); HR-MS (FAB+): calcd for C_34_H_45_O_2_^79^Br [M]^+^*m*/*z* = 564.2603, found *m*/*z*: 564.2628.

### Synthesis of compound **M**

To a mixture of compound **3** (763 mg, 1.35 mmol) and potassium hydroxide (151 mg, 2.78 mmol) in acetonitrile (6 mL) was added imidazole (645 mg, 9.48 mmol). The reaction mixture was refluxed for 8 h and then cooled to room temperature. After a flash column chromatography (ethyl acetate/methanol = 25 : 2), the crude product was further washed with deionized water (15 mL) and dried under vacuum to obtain pure white compound **M** (371 mg, 49.8%). ^1^H NMR (400 MHz, CDCl_3_, 298 K): *δ* = 7.46 (s, 1H), 7.44 (d, *J* = 8.8 Hz, 4H), 7.07 (s, 1H), 6.91 (s, 1H), 6.84 (d, *J* = 8.8 Hz, 2H), 6.81 (d, *J* = 8.4 Hz, 2H), 3.95 (m, 6H), 1.78 (m, 6H), 1.27 (br, 22H), 0.89 (t, *J* = 6.8 Hz, 3H). *δ* = 159.90, 159.68, 137.14, 134.04, 134.04, 129.51, 118.79, 114.67, 114.62, 113.90, 113.63, 81.41, 81.21, 73.02, 72.87, 68.16, 67.73, 46.96, 31.93, 31.03, 29.67, 29.65, 29.60, 29.57, 29.37, 29.37, 29.16, 28.97, 26.33, 26.00, 25.61, 22.71, 14.13. MS (FAB+): calcd for [M + H]^+^*m*/*z* = 553.4, found *m*/*z*: 553.5; HR-MS (FAB+): calcd for C_37_H_49_O_2_N_2_ [M + H]^+^*m*/*z* = 553.3794, found *m*/*z*: 553.3818.

### Synthesis of the block copolymer **P**

P[S(5.4k)-*co*-BrS(0.7k)]-*b*-PAA(8.1k) (**P**) was synthesized by reversible addition–fragmentation chain transfer (RAFT) with 10 mol% incorporation of BrS according to previously reported literature.10*a* The total characterizations of the current block copolymer are: *M*_n_ = 14 200 Da, *M*_w_ = 15 400 Da, *M*_w_/*M*_n_ = 1.08.

### Preparation of the co-micelle **P-M**

Step (1) THF solutions of **P** with concentration of 1 mg mL^–1^ (8 mL) were added dropwise into toluene (8 mL) with stirring. The mixed solution was placed in a 20 mL vial to allow the solvent to evaporate naturally until 2 mL was left. Then toluene (6 mL) was added to the solution for further usage; Step (2) **M** (1.7 mg) was dissolved into a small amount of THF and was dropped into the above-mentioned toluene solution of **P**. Such a system was kept in place and evaporation was allowed naturally until 2 mL was left to form a steady **P-M** micelle. Tiny uncoated **M** was removed by dialysis and the collected co-micelle solutions were kept for the later related measurements.

### Preparation of **P-M** doped PMMA film

The above-mentioned colloidal solution of **P-M** was mixed into a saturated toluene solution of PMMA with the same volume. The solid film was then obtained by drop-casting this mixed solution onto one side of a 1 mm quartz cuvette.

### Transient absorption spectroscopy

The pump laser light (∼100 fs pulse width) comes from an optical parametric amplifier pumped by a Ti:Sapphire femtosecond regenerative amplifier (800 nm, 1 kHz rep-rate). The probe light is a white-light supercontinuum, 450–900 nm. The pump and probe beams overlapped under a small angle. The detection consists of a pair of high-resolution multichannel detector arrays coupled to a high-speed data acquisition system.

## Supplementary Material

Supplementary information
