# Supplementary material for: Sequential oligodiacetylene formation for progressive luminescent color conversion via co-micellar strategy
Source: Chem Sci. 2015 Dec 9;7(3):2058–65. doi: 10.1039/c5sc04253d (PMC5968548; doi:10.1039/c5sc04253d)
Supplement: Supplementary file 1 [file SC-007-C5SC04253D-s001.pdf]

Electronic Supplementary Material (ESI) for Chemical Science.
This journal is © The Royal Society of Chemistry 2015

Electronic Supplementary Information

Sequential Oligodiacetylene Formation for Progressive Luminescent Color Con-version via Co-Micellar Strategy

Liangliang Zhu,*[,a,b] M. Tuan Trinh,[b] Liyuan Yin [a] and Zhiyun Zhang [c]

[a] State Key Laboratory of Molecular Engineering of Polymers,

Department of Macromolecular Science, Fudan University, Shanghai 200433, China.

[b] Department of Chemistry, Columbia University, New York, NY 10027, USA.

[c] Department of Chemistry, National Taiwan University, Taipei 10617, Taiwan.

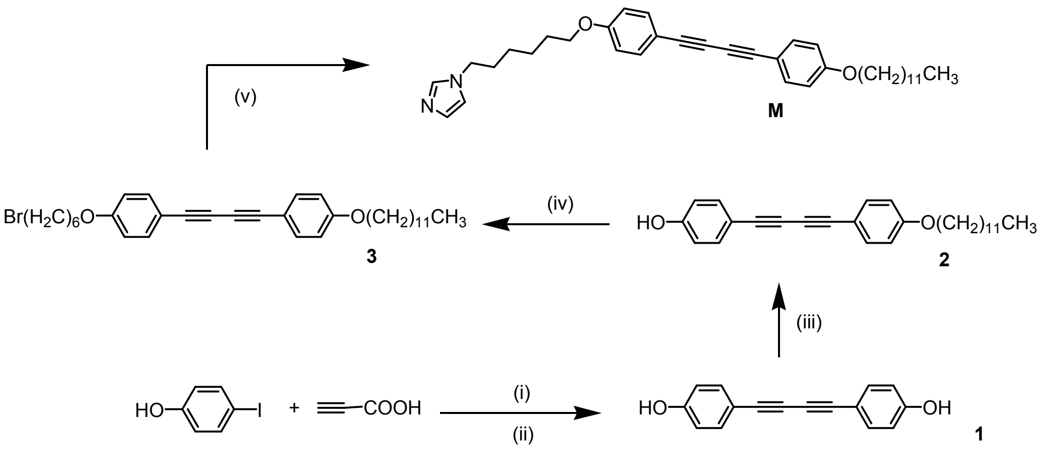

Figure S1. Synthetic route for the preparation of M. (i) $Pd(PPh_3)_2Cl_2$, CuI, Et_3N, DMF, (ii) Ag_2CO_3, heating, (iii) *n*-dodecyl bromide, K_2CO_3, (iv) dibromoalkane, K_2CO_3 and (v) imidazole, KOH.

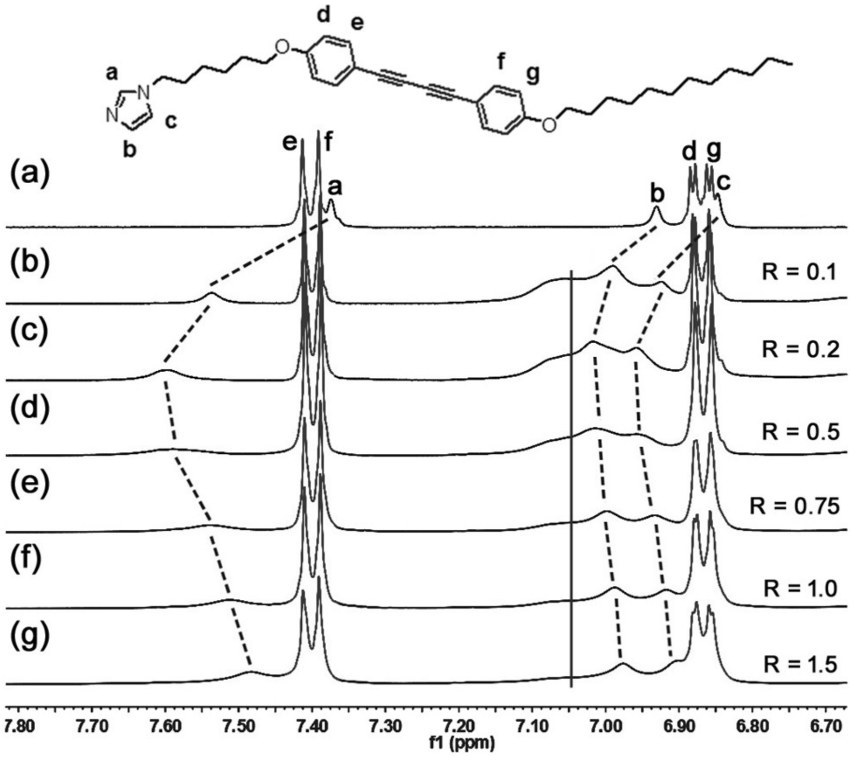

Figure S2. Tuning of the packing interactions by varying the loading molar ratio. Partial ^1H NMR spectra (400 MHz, THF-d_8, 298 K) of (a) the neat M and P-M at R = (b) 0.1, (c) 0.2, (d) 0.5, (e) 0.75, (f) 1.0, and (g) 1.5. R = moles of the M monomer relative to moles of the acrylic acid units.

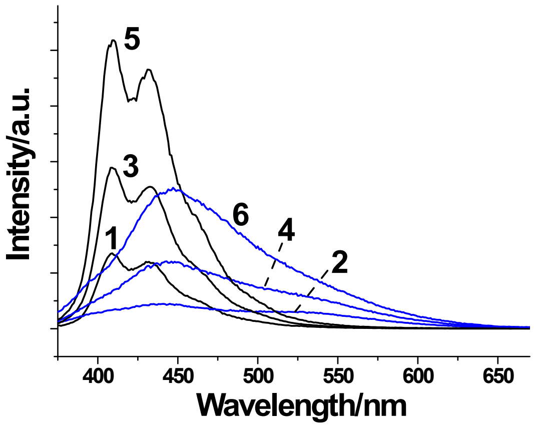

Figure S3. Emission spectra (λ_{ex} = 365 nm) of P-M at different R before (curve 1, 3 and 5 for R = 0.2, 0.4 and 0.9, respectively) and after (curve 2, 4 and 6 for R = 0.2, 0.4 and 0.9, respectively) irradiation at 254 nm for 5 min. The spectra were determined with the concentration of 27 μg/mL calculated from the P in chloroform at room temperature. The generation of the oligodiacetylene emission was weakened at high loading, indicating that more dissociated monomers at higher loading are unfavorable for photocrosslinking.

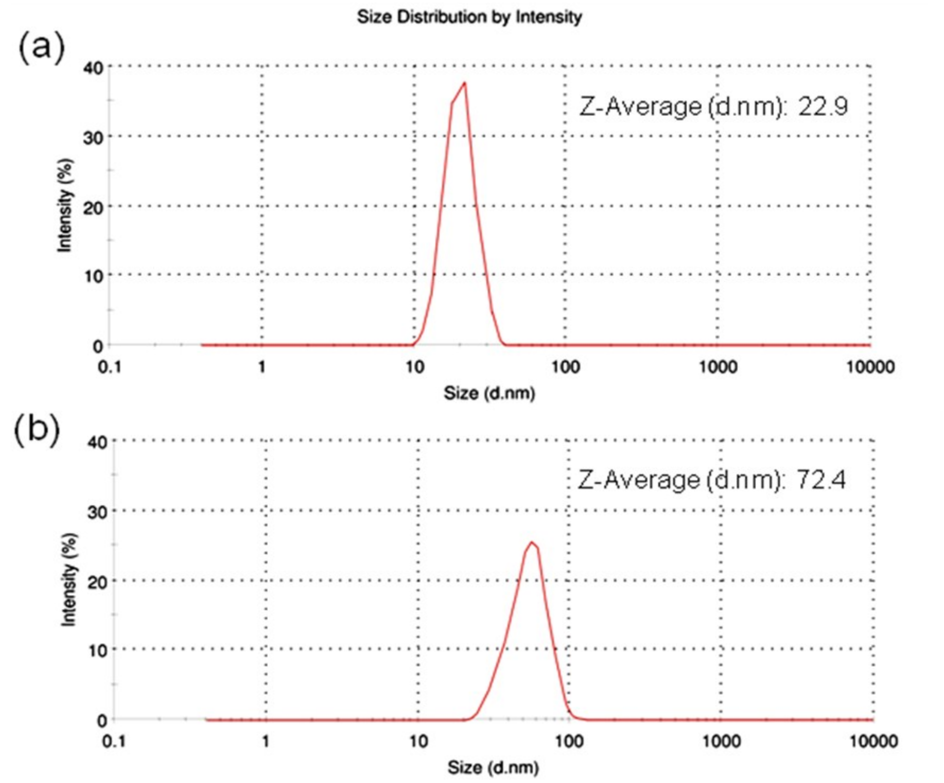

Figure S4. Distribution of the dynamic diameter of (a) P and (b) P-M in toluene at room temperature.

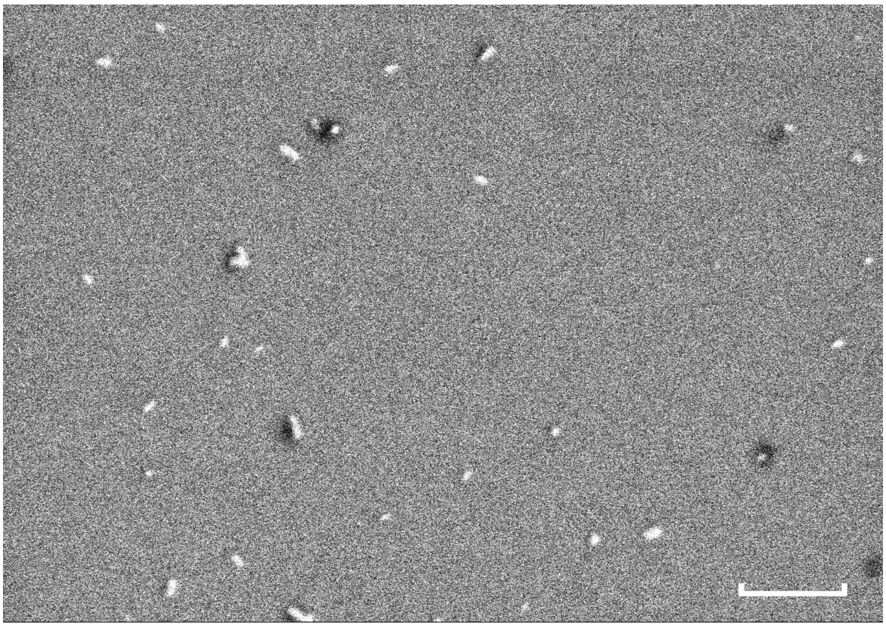

Figure S5. SEM image with a relatively large scale of P-M spun from toluene solution, respectively. Scale bar: 500 nm.

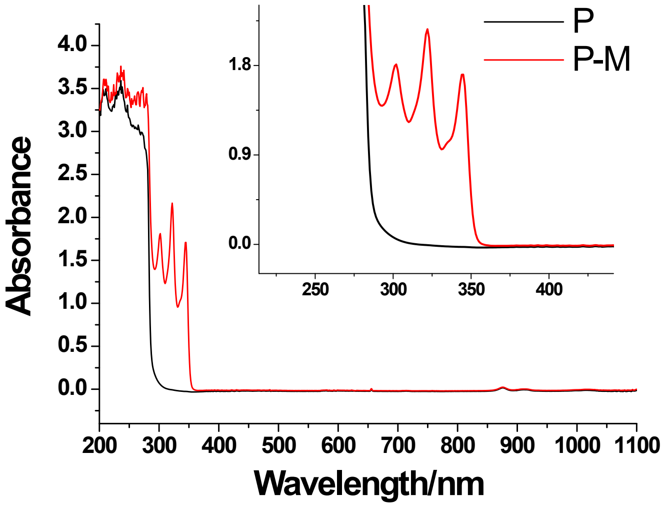

Figure S6. Absorption spectra of P and P-M in toluene at room temperature.

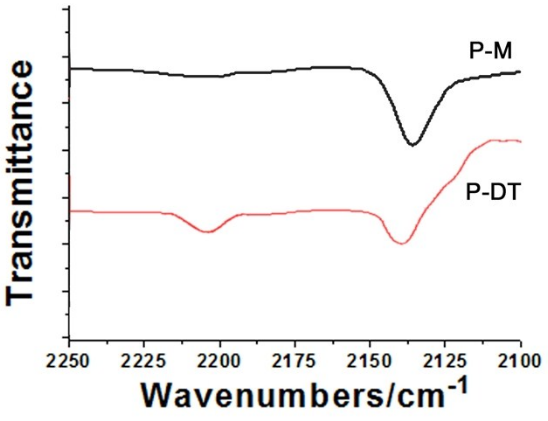

Figure S7. IR spectra of the diacetylene vibration range of P-M and P-DT.

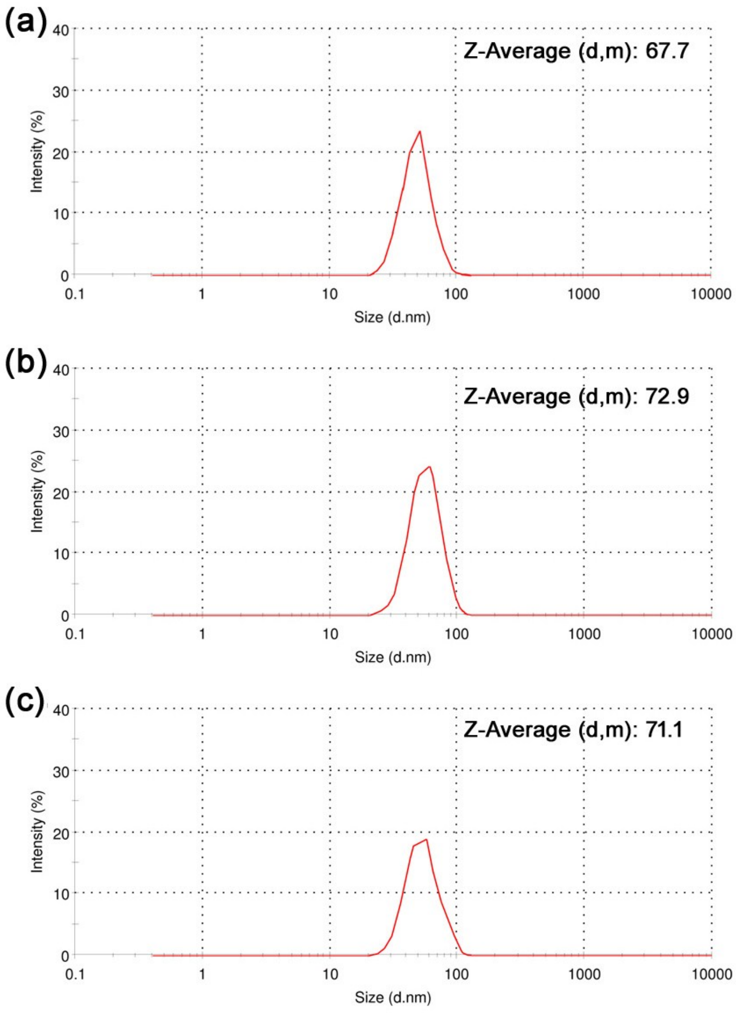

Figure S8. Distribution of the dynamic diameter of (a) P-T, (b) P-DT and (c) P-DT2 in toluene at room temperature.

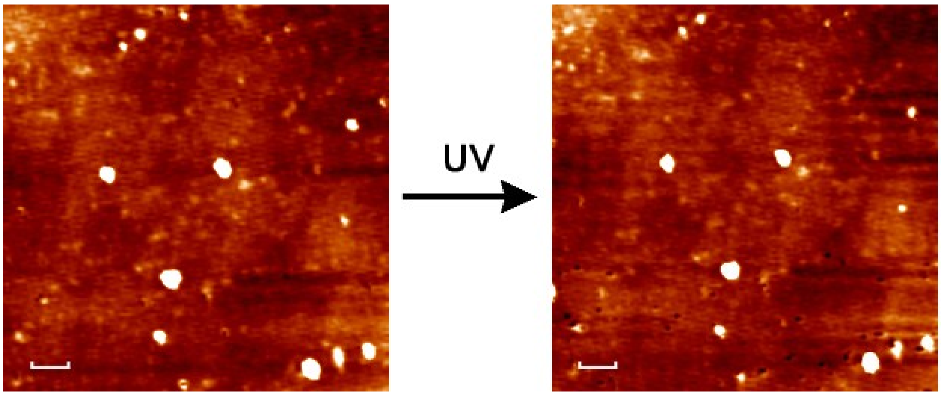

Figure S9. AFM images of P-M spun from toluene solution (left) before and (right) after irradiation at 254 nm for 30 min. The two images were taken at the same location.

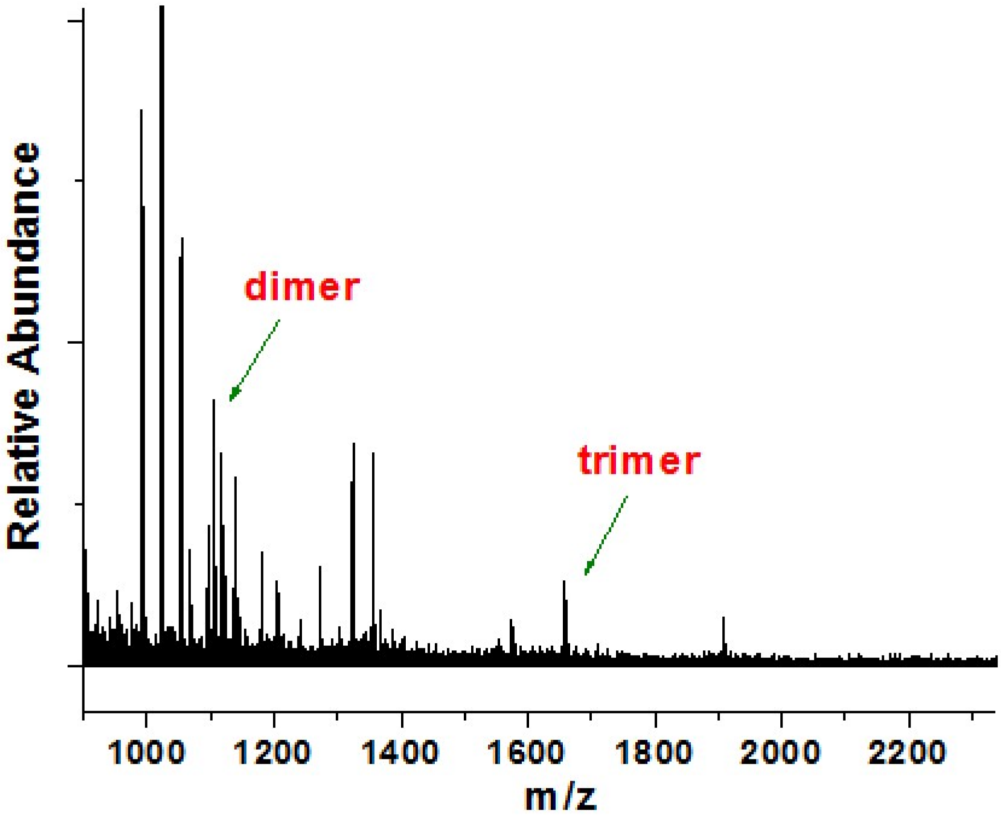

Figure S10. MALDI-TOF MS full spectrum of P-DT, prepared from P-M in toluene after photoirradiation at 254 nm for 30 min.

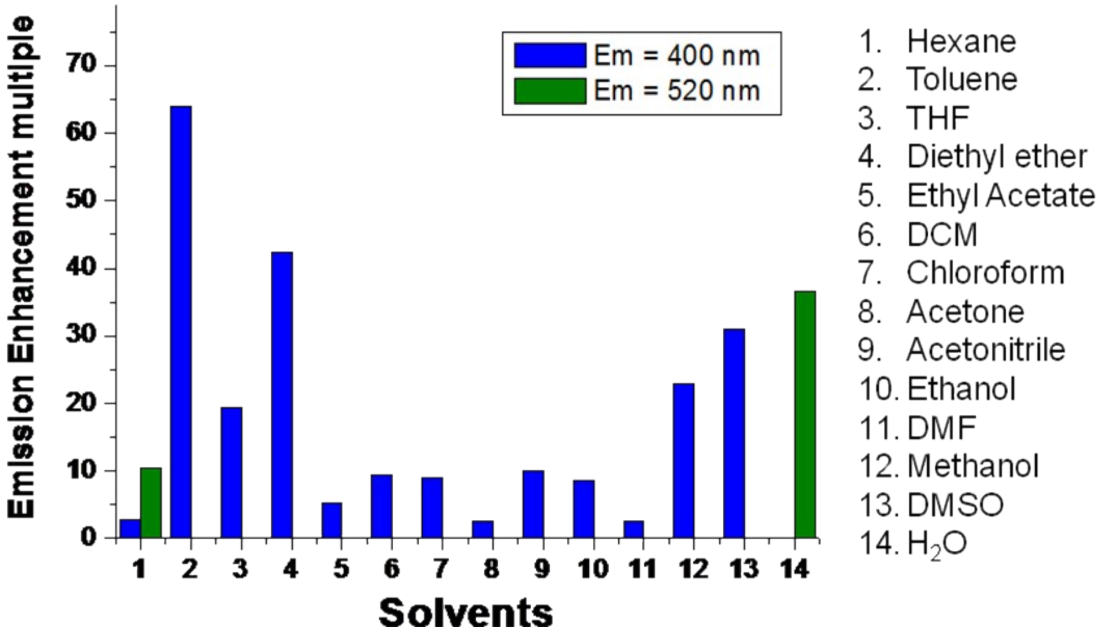

Figure S11. Emission enhancement of M in different solvents after irradiation at 254 nm for 30 min at room temperature. Here the emission enhancement multiple is defined by dividing the emission intensity after irradiation for 30 min by that without irradiation. This figure can reflect the ability of aggregation induced photocrosslinking of M in different solvents. The concentration of M is 40 μM in these solvents.

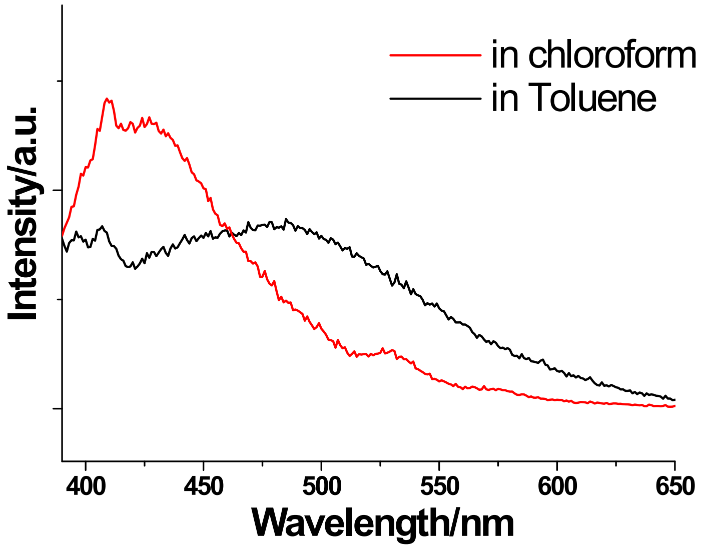

Figure S12. Emission spectra of M in chloroform and in Toluene at room temperature.

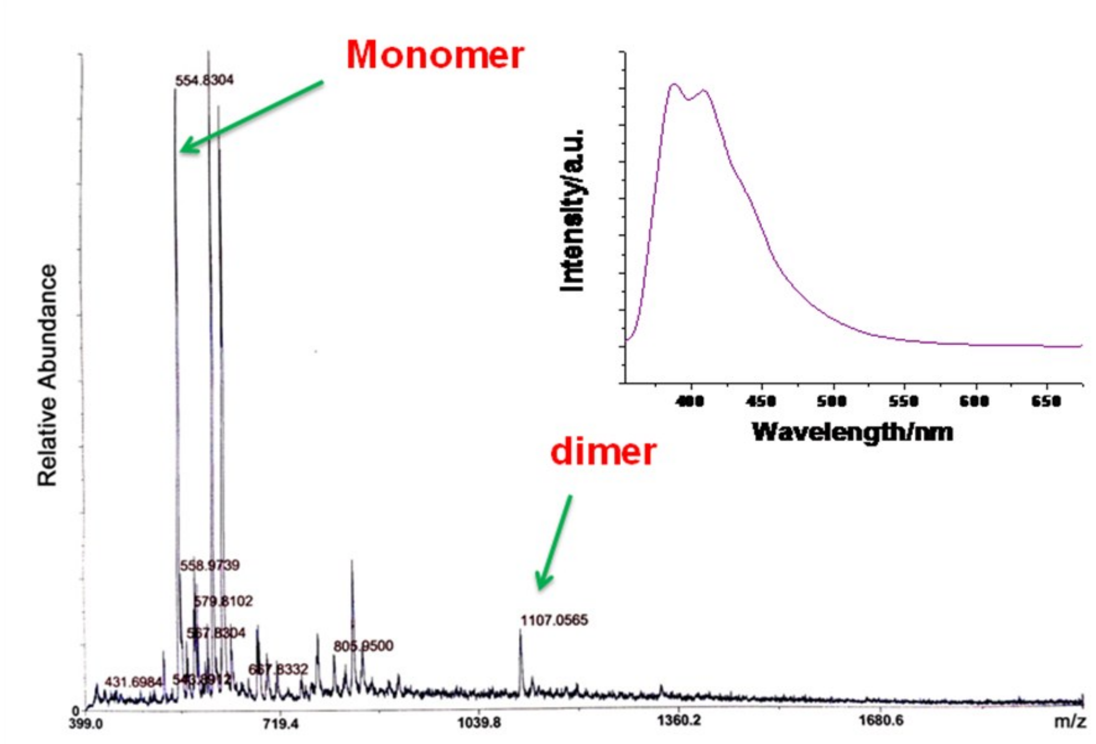

Figure S13. MALDI-TOF MS spectrum of M from toluene solution after irradiation at 254 nm for 30 min. The insert shows the corresponding emission spectrum of the toluene solution of M after irradiation at 254 nm for 30 min.

Table S1. Abundance change of the monomer, dimer and trimer signals in MALDI-TOF MS spectra upon irradiation at 254 nm for different periods. These MS data were collected under the same laser beam.

Time	Relative Abundance [monomer+H]	Normalized Relative Abundance [monomer+H]
0	18250.44532	1
5	13505.32953	0.74
10	9855.24047	0.54
15	6898.668329	0.378
20	5767.14072	0.316
25	4635.61311	0.254
30	4872.868899	0.267
**Time**	**Relative Abundance [dimer+H]**	**Normalized Relative Abundance [dimer+H]**
0	22.67233326	0.046
5	453.939542	0.921
10	483.0192738	0.98
15	491.3981796	0.997
20	495.3411941	1.005
25	490.412426	0.995
30	492.87681	1

Time	Relative Abundance [trimer+H]	Normalized Relative Abundance [trimer+H]
0	2.00492604	0.013
5	50.123151	0.325
10	84.0526686	0.545
15	111.1962827	0.721
20	140.807498	0.913
25	149.1356524	0.967
30	154.22508	1

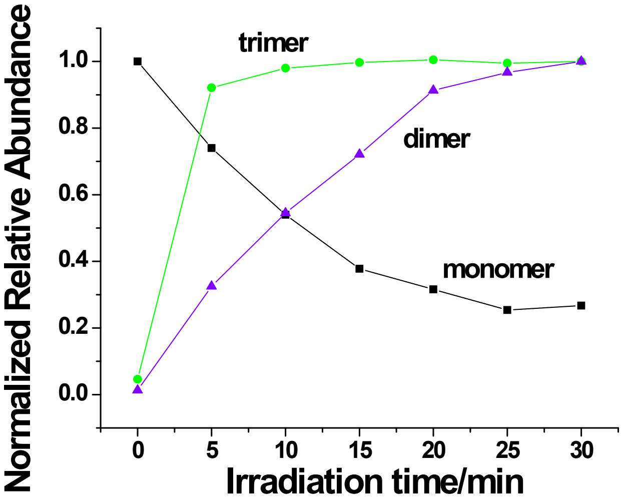

Figure S14. The traces of the Normalized Relative Abundance versus irradiation time from Table S1.

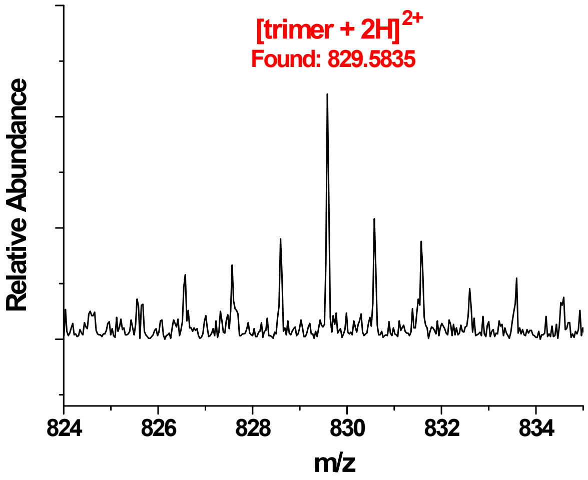

Figure S15. MALDI-TOF MS spectra of P-DT showing the signal of [trimer + 2H]$^{2+}$. This finding further confirmed the molecular structure with two terminal protons alongside the enyne skeleton.

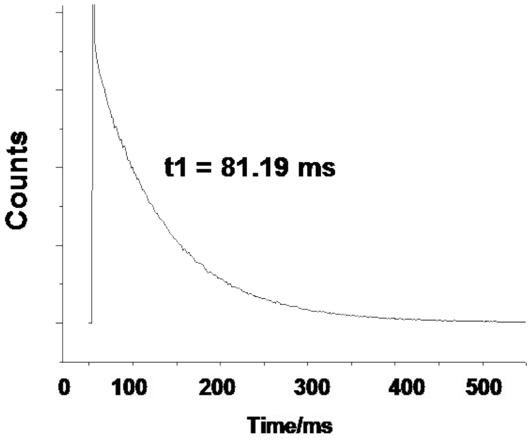

Figure S16. PL lifetime of P-M measured at 500 nm emission monitored upon excitation at 345 nm in toluene at 77 K.

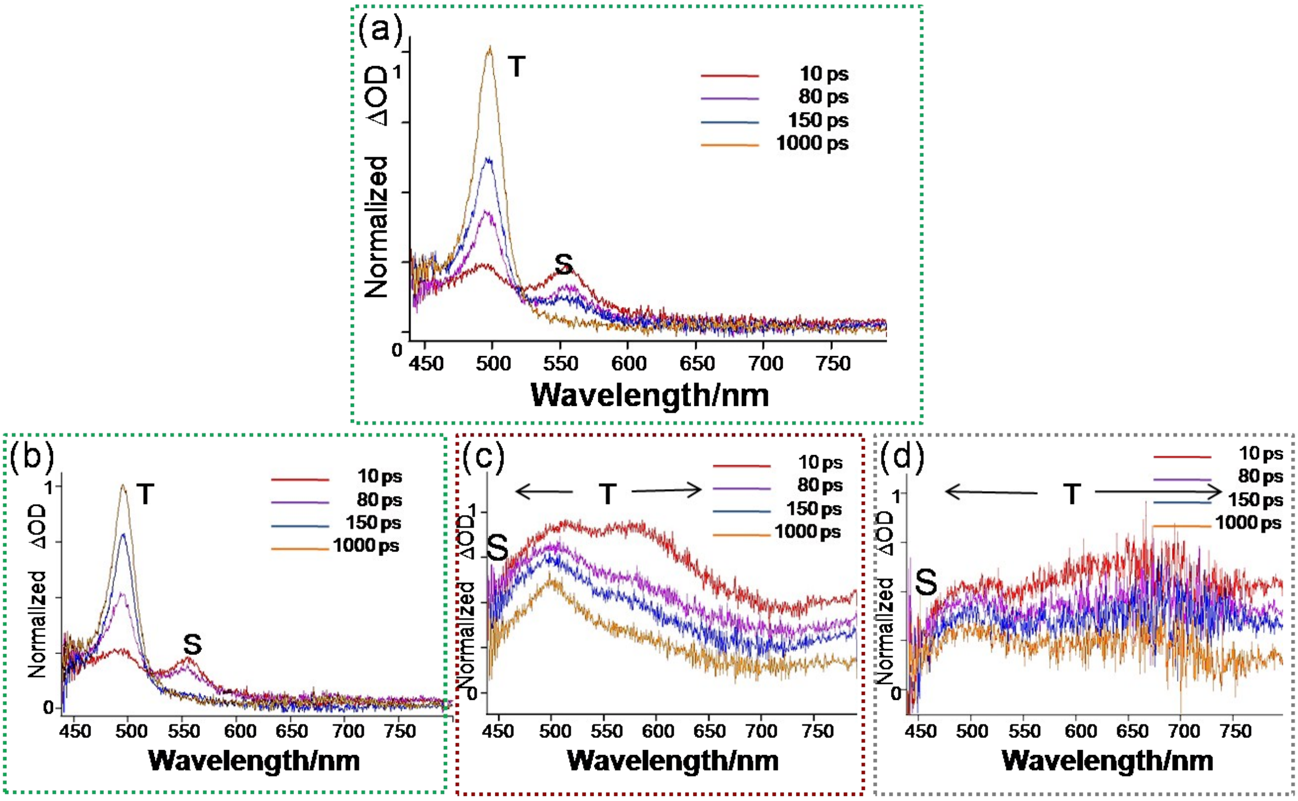

Figure S17. Time independent transient absorption spectral signatures of (a) P-M pumped at 320 nm and of P-DT pumped at (b) 320 nm, (c) 375 nm and (d) 400 nm, respectively, in Toluene at room temperature.

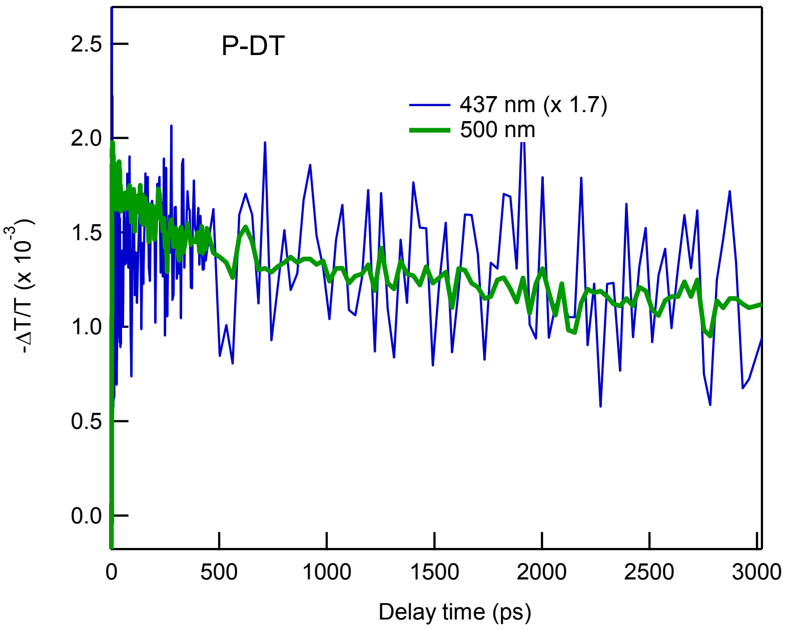

Figure S18. The similarity in dynamics at 437 nm (x 1.7) and 500 nm at a long delay time window for P-DT upon 375 nm excitation (figure 4)

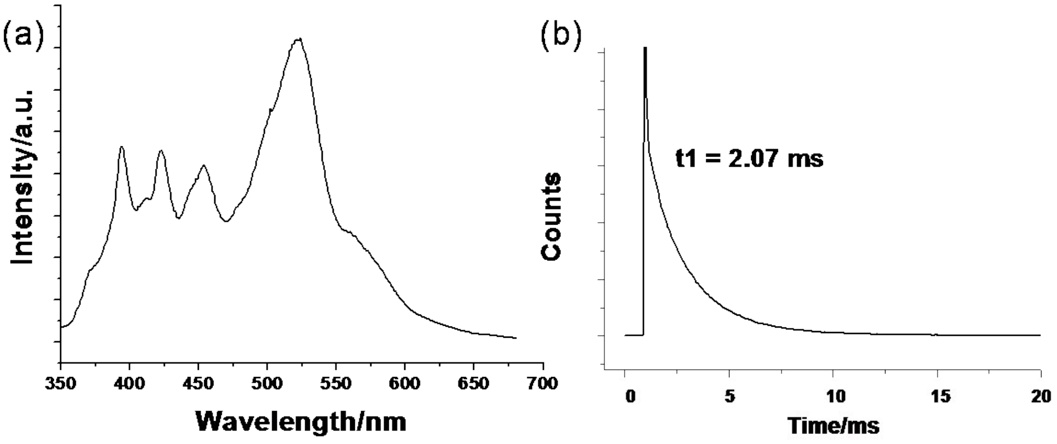

Figure S19. (a) Emission (λ_{ex} = 345 nm) of **P-DT** in toluene at 77 K. (b) PL lifetime of **P-DT** in toluene measured at 522 nm detection wavelength upon 372 nm excitation at 77 K.

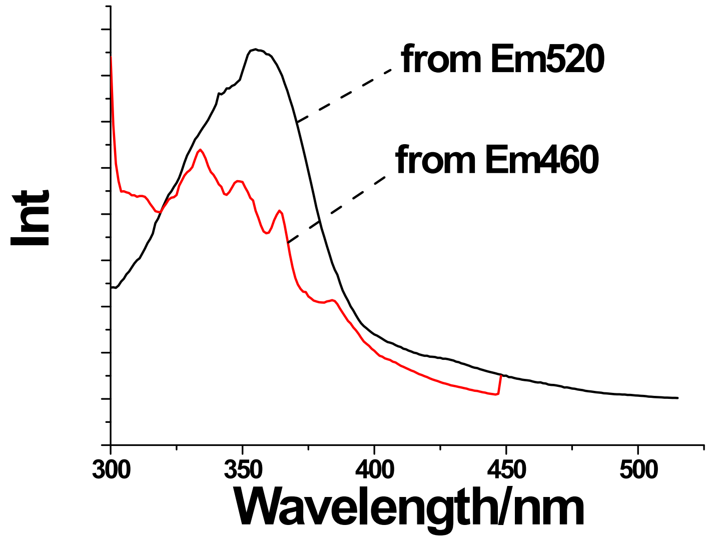

Figure S20. Excitation Spectra of **P-DT** in toluene detected at 460 nm and 520 nm emission wavelengths at 77 K.

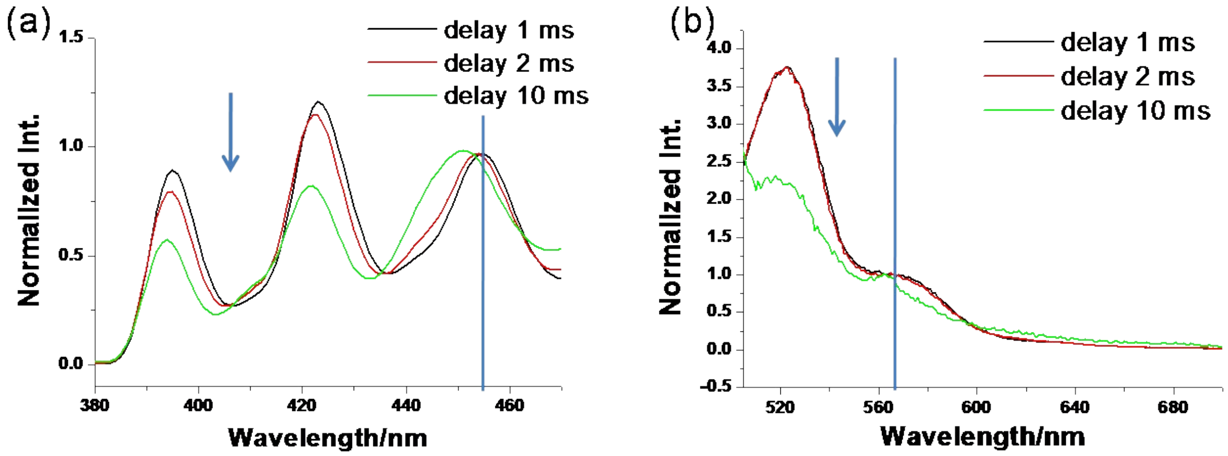

Figure S21. Comparison of emission spectra (λ_{ex} = 345 nm) of **P-DT** in Toluene at room temperature (RT) and at 77 K.

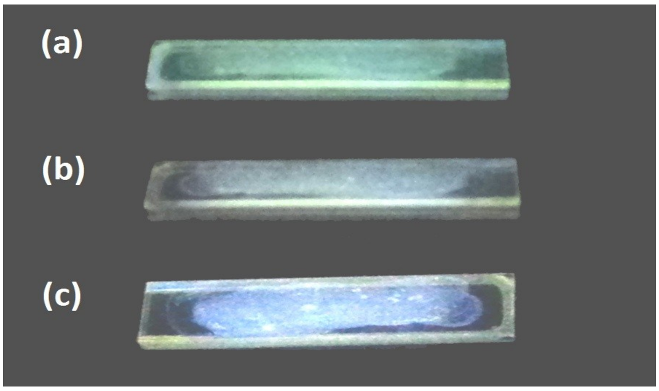

Figure S22. Photographs of **P-M** doped PMMA film after photoirradiation at 254 nm for (a) 5 min, (b) 30 min and (c) 150 min under a UV light (λ = 365 nm).

Luminescent quantum yield was determined in toluene solution using optically matching ethanol solution of Rhodamine B (Φ_r = 0.65) as standard. The quantum yield was calculated using the equation below:

$$\Phi_f = \Phi_r\ (A_rF_s/A_sF_r)\ (\eta_s^2/\eta_r^2)$$

where, A_s and A_r are the absorbance of the sample and reference solutions, respectively, at the same excitation wavelength, F_s and F_r are the corresponding relative integrated emission intensities, and η is the refractive index of the solvent. The excitation wavelength is 365 nm, and we maintained the absorbance at 365 nm <=0.05 during our measurements.
